# Fatal septic shock due to disseminated coccidioidomycosis: a case series and review of the literature

**DOI:** 10.1186/s12879-023-08379-6

**Published:** 2023-06-26

**Authors:** Piotr Wisniewski, Isaac McCool, John C. Walsh, Chelsea Ausman, Jenifer Edmondson, Alexandra Perry, Evan C. Ewers, Ryan C. Maves

**Affiliations:** 1 Operational Infectious Diseases Directorate, Naval Health Research Center, San Diego, CA USA; 2 Uniformed Services University School of Medicine, Bethesda, MD USA; 3 Division of Infectious Diseases, Naval Medical Center San Diego, San Diego, CA USA; 4 Department of Pathology, Naval Medical Center San Diego, San Diego, CA USA; 5 Department of Pulmonary and Critical Care Medicine, Naval Medical Center San Diego, San Diego, CA USA; 6 Department of Medicine, Fort Belvoir Community Hospital, Fort Belvoir, VA USA; 7 Sections of Infectious Diseases and Critical Care Medicine, Wake Forest University School of Medicine, North Carolina Baptist Hospital, 1 Medical Center Boulevard, Winston-Salem, NC 27157 USA

**Keywords:** Coccidioidomycosis, Coccidioides, Sepsis, Septic shock

## Abstract

**Background:**

Coccidioidomycosis is a fungal infection endemic to the southwestern United States and regions of Latin America. Disseminated disease occurs in < 1% of cases. Septic shock is even rarer, with high mortality despite therapy.

**Case summary:**

We describe two cases of coccidioidal septic shock. Both patients were older men of Filipino ancestry presenting with respiratory failure and vasopressor-dependent shock. Antifungal drugs were initiated after failure to improve with empiric antibiotics; in both, *Coccidioides* was isolated from respiratory cultures. Despite aggressive care, both patients ultimately died of their infections. We provide a review of the published literature on this topic.

**Conclusions:**

Most of the 33 reported cases of coccidioidal septic shock occurred in men (88%) of non-white race and ethnicity (78%). The overall mortality rate was 76%. All survivors received amphotericin B as part of their treatment. Coccidioidomycosis-related septic shock is a rare disease with poor outcomes; delays in diagnosis and treatment are common. Improved diagnostic testing for coccidioidomycosis could enhance recognition of this disease in the future. Although data are limited, early treatment with amphotericin B in cases of coccidioidal septic shock may reduce mortality.

## Introduction

Coccidioidomycosis is a fungal infection endemic to the southwestern United States and regions of Latin America [1]. Most infections are confined to the lungs, but disseminated disease occurs in < 1% of cases and leads to significant morbidity and potential mortality [2]. Septic shock due to coccidioidomycosis is a rare with a high associated risk of death. Procalcitonin (PCT) is not typically elevated in non-critically ill patients with coccidioidomycosis [3]; it is unclear if this applies to very sick patients. Here, we describe two cases of coccidioidomycosis complicated by septic shock from our hospital and include a review of the literature.

## Case report 1

**Fig. 1:**
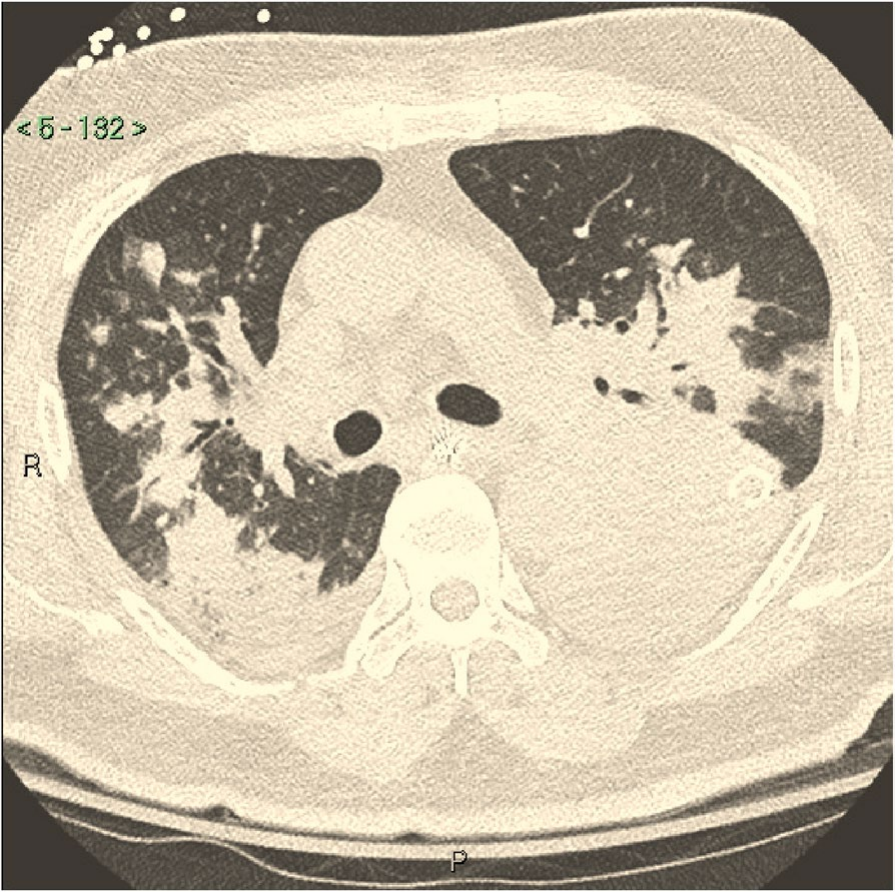
Non-contrast enhanced computed tomography of the chest, patient #1, demonstrating multifocal airspace opacities and a left pleural effusion with a tube thoracostomy in place

A 70-year-old Filipino man with type 2 diabetes mellitus presented to the emergency department (ED) with complaints of dyspnea, cough, and malaise. He resided in southern California and had no recent travel history. After initial evaluation, he was diagnosed with community-acquired pneumonia and was prescribed moxifloxacin 400 mg by mouth daily for an anticipated seven day course. He failed to improve on moxifloxacin and returned to the ED two days later with worsening cough. In the ED, the patient was tachycardic, tachypneic, and hypoxemic. He was admitted to the hospital and received ceftriaxone 1 g intravenously (IV) every 24 h and azithromycin 500 mg daily by mouth. Computed tomography of the chest demonstrated a progressively worsening multilobar pneumonia as well as a left pleural effusion (Fig. 1). Tube thoracostomy was performed, demonstrating an exudative effusion with lymphocytes but no visible organisms on Gram stain.

**Fig. 2:**
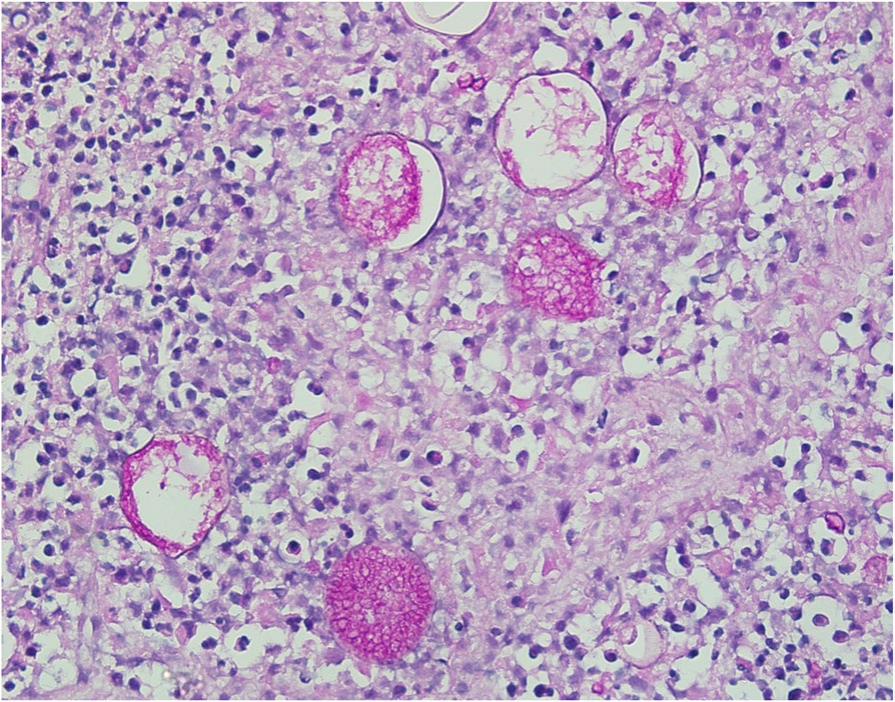
**A** Lung tissue from autopsy, patient #1, 40 × magnification, PAS-D stain, demonstrating the characteristic spherules of *Coccidioides*

On the fourth hospital day, he became increasingly tachypneic and hypoxemic, requiring transfer to the intensive care unit (ICU) where he underwent endotracheal intubation and mechanical ventilation. Despite broad-spectrum antibacterial therapy, his hemodynamics continued to worsen, requiring escalating levels of vasopressor support. Anuria and refractory acidemia developed over the next 48 h, and continuous renal replacement therapy (CRRT) was initiated. Sputum cultures from the time of intubation demonstrated small fungal colonies. On the sixth hospital day, serologic testing for *Coccidioides* returned as positive, and fluconazole 800 mg IV daily and caspofungin 70 mg IV daily were started. The aforementioned sputum isolate was ultimately confirmed as *Coccidioides immitis*. The patient’s oxygenation and hemodynamics initially improved on antifungal therapy. On the ninth hospital day, however, he developed ventricular fibrillation and cardiac arrest; attempts at resuscitation were unsuccessful, and he died thereafter,

A postmortem examination showed a diffuse, thick white purulence present throughout the lungs bilaterally, most significantly involving the left lower lobe. Microscopic examination of the lung tissue demonstrated necrosis and inflammation, as well as *Coccidioides* spherules and spores (Fig. 2). Additionally, spherules were identified microscopically in the liver and spleen.

## Case report 2

**Fig. 3:**
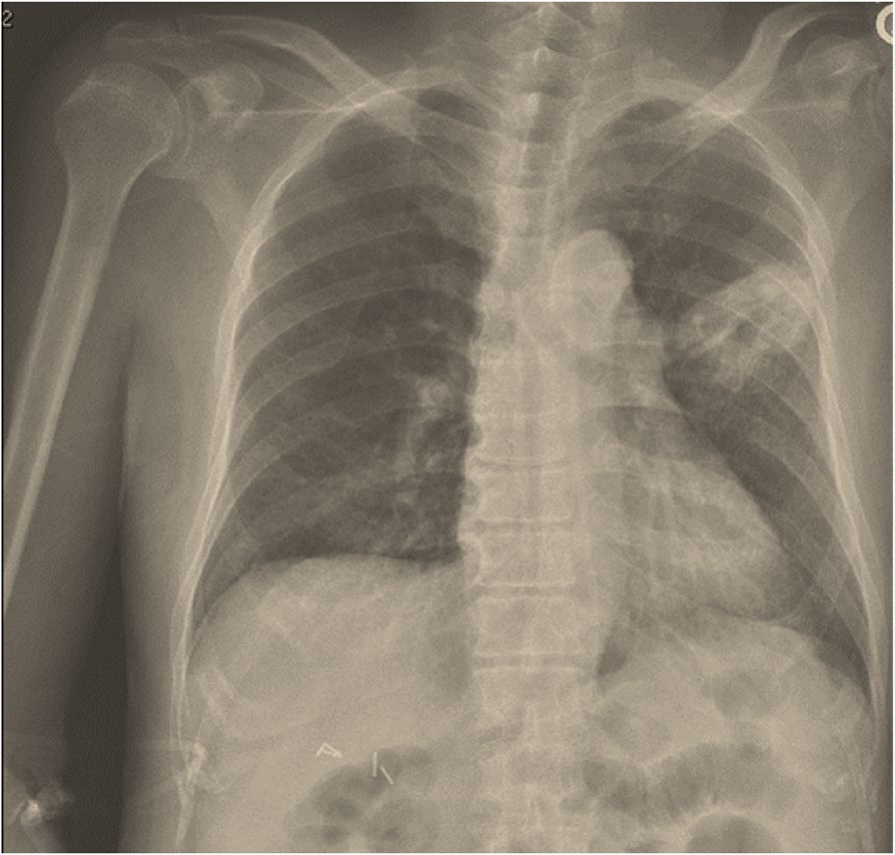
Anteroposterior radiograph of the chest, patient #2, demonstrating a cavitary lesion in the left upper lobe

A 74-year-old Filipino man developed a dry cough 3 weeks prior to presentation. He had no overt history of desert or dust exposure other than residence in southern California. He was treated with a 5-day course of oral azithromycin without improvement. Subsequently, his family noted a progressive decline in his alertness and energy level, accompanied by intermittent low-grade fevers with poor appetite and weight loss. Two days prior to presentation, the patient’s cognitive function declined dramatically. He stopped responding to questions and became incontinent of urine. He was brought to the ED by his family, where he was febrile at 39.1 °C, tachycardic (134 beats/minute), tachypneic (32 breaths/minute), but normotensive (123/70 mmHg). A physical examination was notable for inspiratory crackles in the left middle lung field. The patient’s initial PCT was 1.73 µg/L (normal range < 0.25 µg/L). Chest radiography demonstrated a cavitary lesion in the left upper lobe (Fig. 3). He was admitted to the ICU; vancomycin 15 mg/kg IV every 12 h and piperacillin-tazobactam 4.5 g IV every eight hours via extended infusion were administered empirically.

His condition worsened on the second hospital day, requiring vasopressor support and endotracheal intubation. He developed an absolute peripheral eosinophil count of 6400 cells/mL and a rising PCT of 2.4 µg/L. His shock worsened, with the development of acute kidney injury requiring CRRT. PCT increased further to 400 µg/L. Culture of bronchoalveolar lavage fluid was notable for yeast forms. The patient was first started on fluconazole 800 mg IV daily for empiric treatment of coccidioidal pneumonia and then switched quickly to liposomal amphotericin B 5 mg/kg IV every 24 h. Unfortunately, he failed to improve over the next 2 days and, after a family discussion, was compassionately extubated on hospital day 6. Following his death, the bronchoalveolar lavage cultures were finalized as *Coccidioides immitis*; his anti-*Coccidioides* complement fixation titer was positive at 1:4.

## Literature review and discussion

We performed a MEDLINE and Google Scholar search using the terms “coccidioidomycosis”, “*Coccidioides*”, “sepsis”, and “septic shock”. English and Spanish-language articles and conference abstracts were included. Manuscripts so identified were then reviewed by the authors. Cases were included if the patient had a positive confirmed diagnosis of coccidioidomycosis (based on culture, serology, or histopathology) and vasopressor-dependent shock without a compelling alternate cause (e.g., concomitant bacteremia). Data on patient age, gender, ethnicity, comorbidities, antifungal and immunomodulatory therapy administered, and outcome were tabulated. We also screened the identified manuscripts for reported PCT values.

Written informed consent was obtained from the families of the two patients included in this report. This review was approved by the Institutional Review Board at Naval Medical Center San Diego in accordance with all relevant U.S. Federal regulations for the conduct of biomedical research. None of the authors reported any potential conflicts of interest in relation to the current work. No funding was received for this work.

We identified 20 reports describing 33 patients with vasopressor-dependent septic shock due to *Coccidioides* infection since 1993 (Table 1), including the 2 patients described here. Of these patients, 29/33 (88%) were male, and 25/33 (76%) died from their illnesses. Of those with ethnicity, 12/26 (46%) were Latino, 6/26 (23%) of African descent, 5/26 (19%) Asian-Pacific Islander, and 3/26 White (12%). All but 2 were adults, with a median age of 58 years. Of the 33 patients, 24 (73%) had reported comorbid conditions, including human immunodeficiency virus (HIV) infection (5/24), solid organ transplantation (4/24), and diabetes mellitus (6/24).

**Table 1:** Demographics, comorbid conditions, therapies, and outcomes of published cases of septic shock associated with coccidioidomycosis

Age	Gender	Race/Ethnicity	Comorbid Conditions	Antifungal Therapy	Outcome	Reference
70	M	Unknown	None reported	Amphotericin B	Died	Lopez et al., 1993 [4]
65	M	Unknown	COPD	None	Died	Lopez et al., 1993 [4]
61	M	Latino	None reported	Amphotericin B	Died	Arsura et al., 1998 [5]
53	M	Latino	Diabetes mellitus	Amphotericin B	Died	Arsura et al., 1998 [5]
58	M	Latino	Alcoholic liver disease	Amphotericin B	Died	Arsura et al., 1998 [5]
54	M	Filipino	None reported	Amphotericin B	Died	Arsura et al., 1998 [5]
40	F	Black	Sarcoidosis, sickle cell trait	Amphotericin B	Died	Arsura et al., 1998 [5]
52	M	Latino	None reported	Amphotericin B	Died	Arsura et al., 1998 [5]
64	F	Filipina	Diabetes mellitus	Amphotericin B	Died	Arsura et al., 1998 [5]
92	M	Latino	None reported	Amphotericin B	Died	Arsura et al., 1998 [5]
47	M	Asian	Kidney transplantation	Amphotericin B	Died	Cha et al., 2000 [6]
23	M	Black	None reported	Amphotericin B	Survived	Shibli et al., 2002 [7]
73	M	Unknown	Constrictive pericarditis	Amphotericin B, fluconazole	Survived	Visbal et al., 2003 [8]
78	M	Unknown	Constrictive pericarditis, CAD, non-Hodgkin lymphoma	Fluconazole	Died	Visbal et al., 2003 [8]
59	M	White	Diabetes mellitus, CAD, COPD	Amphotericin B, APC	Survived	Crum et al., 2004 [9]
76	M	White	None reported	Amphotericin B, APC	Survived	Crum et al., 2004 [9]
36	M	White	HIV/AIDS	None	Died	Rempe et al., 2007 [10]
61	M	“Middle Eastern”	None reported	None	Died	Rempe et al., 2007 [10]
46	F	Unknown	HIV/AIDS	None	Died	Rempe et al., 2007 [10]
25	M	Latino	HIV/AIDS	Amphotericin B, voriconazole	Died	Desai et al., 2010 [11]
34	M	Latino	HIV/AIDS	Voriconazole, caspofungin	Died	Desai et al., 2010 [11]
23	M	Latino	Kidney, liver transplantation	Amphotericin B, caspofungin	Survived	Blodget et al., 2011 [12]
13	M	Unknown	Hemophagocytic lymphohistiocytosis	None	Died	Ramsi et al., 2014 [13]
5	M	Black	None reported	None	Died	El Dib et al., 2014 [14]
54	M	Latino	Cirrhosis, splenectomy	Amphotericin B, fluconazole	Died	Sinha, et al., 2018 [15]
70	M	Filipino	Diabetes mellitus	Fluconazole, caspofungin	Died	Current publication
38	M	Latino	Asthma	Amphotericin B, fluconazole, voriconazole	Survived	Eltayeb et al., 2019 [16]
65	F	Unknown	SLE, ESRD	Micafungin	Died	Berenji et al., 2019 [17]
61	M	Black	Diabetes mellitus, hypertension, distant smoking history	Fluconazole, amphotericin B	Survived	Chang et al., 2019 [18]
69	M	Latino	Hypertension	Fluconazole	Died	Gulati et al., 2019 [19]
31	M	Asian	Henoch–Schönlein purpura	Amphotericin B, fluconazole, micafungin, posaconazole	Survived	Tandon et al., 2020 [20]
61	M	Latino	HIV/AIDS	None	Died	Aduroja et al., 2021 [21]
74	M	Filipino	Diabetes mellitus	Fluconazole, amphotericin B	Died	Current publication

*AIDS* Acquired immunodeficiency syndrome, *APC* Activated protein C, *CAD* Coronary artery disease, *COPD* Chronic obstructive pulmonary disease, *ESRD* End-stage renal disease, *HIV* Human immunodeficiency virus, *SLE* Systemic lupus erythematosus

Mortality was high across all reported cases. Of note, 22 patients received amphotericin B, including all of the survivors. Among those patients treated with amphotericin B, 13/22 died compared with 12/12 treated with other agents or not treated at all. Of the 3 cases with reported PCT levels, all were elevated (Wisniewski: > 400 µg/L; Aduroja: 6.1 µg/L; Berenji: > 20 µg/L).

The two cases and prior reported cases suggest several key points for the management of patients with *Coccidioides* sepsis. *Coccidioides* sepsis is rare but may be underdiagnosed because its rarity precluded clinicians from suspecting the diagnosis. As such, clinicians must maintain a degree of suspicion if a patient has been exposed to an endemic area, especially with risk factors for complicated disease. A clinical trial of fluconazole as a component of therapy for acute community-acquired pneumonia in coccidioidomycosis-endemic regions was halted due to slow enrollment, limiting our ability to assess the impact of early antifungal therapy in patients at risk for severe disease [22]. Regardless, diagnostic evaluation for *Coccidioides*, including serologic testing and careful examination of respiratory cultures, seems prudent for at-risk patients who are not improving on empiric antibacterial therapy. Recent years have seen an expansion in the geographic distribution of coccidioidomycosis in North America, with locally-acquired infections identified outside of its historic endemic zones, suggesting that clinicians may need to maintain a higher index of suspicion [23]. Increased use of sputum Gram stain and culture in patients presenting to EDs with community-acquired pneumonia may be useful in helping identify seriously-ill patients more rapidly, given that early coccidioidal growth may be seen within 2–3 days.

*Coccidioides* septic shock carries a high mortality risk, with as many as 3 of every 4 patients succumbing to the disease. Though this could be due to multiple factors, delays in diagnosis and early aggressive therapy are likely among them. In the 33 patients in the literature, treatment with amphotericin B appears to correlate with better survival. Although no high-quality evidence exists on this topic, the 2016 Infectious Diseases Society of America guideline for the management of coccidioidomycosis recommends the use of amphotericin B in severe disease [24]. Liposomal amphotericin B (LAmB) was approved for use in the United States by the Food and Drug Administration (FDA) in 1997; patients treated prior to 1997 in our case series presumably received amphotericin B deoxycholate, whereas cases treated later received predominantly lipid formulations (LAmB or amphotericin B lipid complex). There are insufficient data to state whether lipid formulations of amphotericin B have an advantage in treating severe coccidioidomycosis. We acknowledge the risk of publication bias in the reported mortality of this series, although the overall mortality of coccidioidal sepsis is likely very high regardless.

We caution against reliance on PCT to exclude coccidioidomycosis in a patient in septic shock. In a 2014 study of 20 patients with coccidioidomycosis, none of whom were critically ill, the median PCT level was 0.05 µg/L [3]. Although these data suggest that PCT elevations are not a typical feature of *Coccidioides* infection, this may not be the case for the minority of patients presenting with vasopressor-dependent shock. Elevated serum PCT levels may reflect severity of illness rather than serve as a differential marker of bacterial versus nonbacterial infections. This phenomenon has been observed other severe nonbacterial diseases, to include COVID-19, influenza, and candidemia [25–28]. As such, PCT may not provide sufficient specificity to determine bacterial versus non-bacterial diseases in life-threatening infections, including coccidioidomycosis.

## Conclusions

Septic shock due to *Coccidioides* infection carries a high risk of death. Delay in diagnosis is common, due to the rarity of the disease and the challenges inherent to its diagnosis. High procalcitonin levels appear to occur in coccidioidal sepsis and may reflect severity of illness, rather than suggesting more common diagnoses such as bacterial sepsis. Improved diagnostic testing for coccidioidomycosis could enhance recognition of this disease in the future. Although data are limited, early treatment with amphotericin B in cases of coccidioidal septic shock may reduce mortality.

## Data Availability

The datasets used and/or analysed during the current study available from the corresponding author on reasonable request.

## Abbreviations

COVID-19: Coronavirus disease 2019
CRRT: Continuous renal replacement therapy
ED: Emergency department
FDA: Food and Drug Administration (United States)
ICU: Intensive care unit
IV: Intravenous
LAmB: Liposomal amphotericin B
PCT: Procalcitonin
